# Nanowaveguide-illuminated fluorescence correlation spectroscopy for single molecule studies

**DOI:** 10.1063/5.0051679

**Published:** 2021-06-04

**Authors:** Joseph M. Chandler, Huizhong Xu

**Affiliations:** Department of Physics and Astronomy, San Francisco State University, 1600 Holloway Avenue, San Francisco, California 94132, USA

## Abstract

Fluorescence Correlation Spectroscopy (FCS) is a method of investigating concentration fluctuations of fluorescent particles typically in the nM range as a result of its femtoliter-sized sample volume. However, biological processes on cell membranes that involve molecules in the μM concentration range require sample volumes well below the conventional FCS limit as well as nanoscale confinement in the longitudinal direction. In this study, we show that an effective measurement volume down to the zeptoliter range can be achieved via the introduction of a nanowire waveguide, resulting in an illumination spot of about 50 nm in lateral dimensions and a longitudinal confinement of around 20 nm just above the waveguide exit surface. Using illumination profiles obtained from finite element method simulations of dielectric nanowaveguides, we perform Monte Carlo simulations of fluorescence fluctuations for two scenarios of fluorophore movement: fluorophores freely diffusing in the three-dimensional (3D) space above the nanowaveguide and fluorophores moving in a two-dimensional (2D) membrane situated directly above the nanowaveguide exit surface. We have developed analytical functions to fit the simulation results and found that an effective illumination size of about 150 zl and 4 × 10^−3^ *µ*m^2^ can be obtained for the 3D and 2D scenarios, respectively. Given the flat surface geometry and the deep-subwavelength confinement of its illumination spot, this nanowaveguide-illuminated fluorescence correlation spectroscopy technique may be well suited for studying the concentration and dynamics of densely distributed protein molecules on cell membranes.

## I. INTRODUCTION

Biological processes occurring between water soluble molecules and cell membranes play important roles in the function of a cell, making membrane proteins a majority of therapeutic targets.^1,2^ To monitor these processes, it is often necessary to investigate the concentrations and dynamics of biomolecules at the interface of the cell surface and solution.^3^ Whereas most traditional biochemical methods for quantifying molecular concentrations destroy the fragile cellular environment being studied, alternative methods such as fluorescence correlation spectroscopy (FCS)^4,5^ have been developed to non-invasively study these concentration fluctuations. FCS uses an objective lens to focus a laser beam to a diffraction-limited spot where fluorophores are excited and then collects that fluorescence signal using the same objective lens.^6^ Using this fluorescence signal, which fluctuates with the number of molecules in the illumination volume, researchers can determine concentration fluctuations and from that determine a variety of properties including diffusion constants and molecule sizes. Additionally, FCS is attractive because it can take instantaneous measurements and can be used with a wide variety of target fluorophores based on the laser wavelength used.^7^

While FCS has been increasingly applied in *in vivo* studies,^8–10^ it suffers from limitations due to its diffraction-limited illumination volume of about 1 fl. This makes traditional FCS effective for concentrations in the nM range; however, the technique becomes inadequate for studying physiologically relevant processes with concentrations in the 1–100 *µ*M range. Furthermore, traditional FCS lacks the required longitudinal confinement needed for studying the dynamics of biomolecules on cell membranes without introducing a background signal from fluorescent molecules residing in the cytosol. While novel detection equipment has been used in recent years to tolerate photon count rates up to tens of MHz and measure correlation curves for sample concentrations in the μM range,^11,12^ the employment of such advanced detection equipment alone will not be adequate when nanoscale information of molecular dynamics in a localized sample region is desired. To overcome these limitations, several approaches have been proposed to increase the illumination confinement and thus reduce the sample volume.^3,13–21^ Among them, total internal reflection fluorescence correlation spectroscopy (TIR-FCS)^3,13–16^ where fluorophores in a solution are excited by an evanescent field generated from total internal reflection of a laser beam enables axial confinement on the order of 200 nm.^15^ However, the lateral confinement in TIR-FCS results from a confocal pinhole in the intermediate image plane of the microscope; therefore, TIR-FCS is still diffraction-limited. On the other hand, STED-FCS^17^ has been developed by combining FCS with super-resolution stimulated emission depletion (STED) microscopy and has been applied to study nanoscale dynamics of molecules in cell membranes.^18–20^ Despite the sub-50-nm lateral resolution, STED-FCS has limitations due to its inadequate confinement in the longitudinal direction and requirements on the photophysical properties of fluorescent labels.^21^

In this study, we propose to use a nanowire waveguide device as the illumination source for fluorescence correlation spectroscopy studies in a process known as nanowaveguide-illuminated fluorescence correlation spectroscopy (NIFS). The technique is based on strongly confined near fields coming out of a nanowire waveguide with resonant transmission characteristics.^22,23^ As a result, the NIFS technique enables confinement of the illumination volume well below the diffraction limit and reduces the FCS sample volume by four orders of magnitude. The flat exit surface of a nanowire waveguide also provides a convenient geometry for the study of molecules confined on a 2D plane such as the cell membrane. Furthermore, the strong longitudinal confinement achieved in NIFS allows us to reduce the background signal from fluorescent molecules residing in the cytosol when studying membrane proteins. We have derived analytical expressions for the autocorrelation functions (ACFs) for NIFS studies of 3D and 2D samples and validated them using Monte Carlo simulations. As the NIFS technique allows for the single-molecule study of biomolecules at physiologically relevant concentrations, it may be well suited for studying the dynamics of densely distributed protein molecules on cell membranes and high-throughput screening of drug candidates for therapeutics.

## II. METHODS

### A. Traditional FCS

The use of the FCS technique centers around fitting the measured autocorrelation function (ACF) with a known analytical form of the function. The ACF, commonly denoted as G(*τ*), is found by time-averaging the product of the deviations in the fluorescence signal from its average (*δF*) at two different times of a fixed delay *τ* and then normalizing it by the square of the average fluorescence signal,^5^

$$G\left(\tau \right)=\frac{\left\langle \delta F\left(t\right)\delta F\left(t+\tau \right)\right\rangle }{{\left\langle F\left(t\right)\right\rangle }^{2}}.$$ (1)

The ACF can be calculated if one knows the density autocorrelation function and the focal illumination profile $W\left(r\right)$,

$$G\left(\tau \right)=\frac{\int \int W\left(r\right)W\left(r^{\prime }\right)\left\langle \delta C\left(r,0\right)\delta C\left(r^{\prime },\tau \right)\right\rangle dVdV^{\prime }}{{\left(\left\langle C\right\rangle \int W\left(r\right)dV\right)}^{2}}.$$ (2)

For classical FCS, the illumination profile of the focal volume $W\left(r\right)$ can be approximated as a Gaussian ellipsoid,

$$W_{FCS}\left(r\right)=e^{-2\left(x^{2}+y^{2}\right)/r_{0}^{2}}\cdot e^{-2z^{2}/z_{0}^{2}}.$$ (3)

Using this profile and the density autocorrelation function for 3D free diffusion, one can obtain the ACF for traditional FCS,

$$G\left(\tau \right)=\frac{1}{V_{e\mathrm{ff}}\left\langle C\right\rangle }{\left(1+\frac{\tau }{\tau_{D}}\right)}^{-1}{\left(1+\frac{\tau }{\kappa^{2}\tau_{D}}\right)}^{-\frac{1}{2}},$$ (4)

where *τ* is the time delay, *V*_eff_ is the effective focal volume and is equal to $\pi^{3/2}r_{0}^{2}z_{0}$, *τ*_*D*_ is the characteristic diffusion time and is given by $\tau_{D}=\frac{r_{0}^{2}}{4D}$, and $\kappa =\frac{z_{0}}{r_{0}}$ is the ratio that determines the oblateness of the spheroidal Gaussian laser focus. By fitting a measured ACF with the analytical ACF given by Eq. (4), one can find the average number of molecules in the focal volume $\left\langle N\right\rangle =V_{e\mathrm{ff}}\left\langle C\right\rangle$, the characteristic diffusion time *τ*_*D*_, and the oblateness of the spheroidal Gaussian focus *κ*.

### B. Nanowaveguide-illuminated fluorescence correlation spectroscopy (NIFS)

**FIG. 1.**
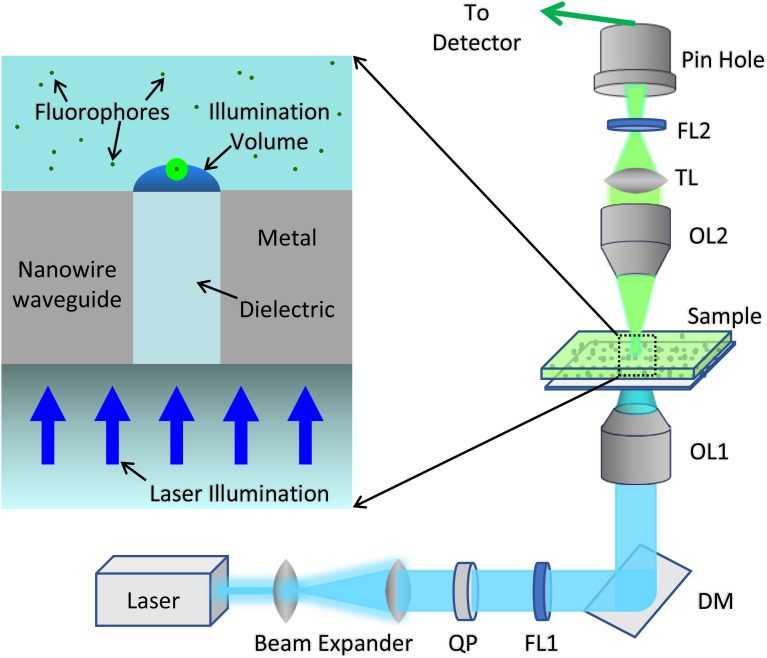
Schematic of the NIFS setup. A circularly polarized laser beam is focused onto a dielectric nanowire waveguide, which also serves as the substrate for a solution of fluorophores. The emitted fluorescence is collected by a top objective conjugate to the focus of the bottom objective. QP: quarter-wave plate, FL1: excitation filter, DM: dichromatic mirror, OL1: bottom objective lens, OL2: top objective lens, TL: tube lens, and FL2: emission filter. The inset shows a close-up view of the nanowire waveguide device.

The proposed NIFS is based on a nanowire waveguide device created by embedding a nanometer-sized cylindrical dielectric nanowire into a metal medium. As shown in Fig. 1, the nanowire waveguide is illuminated from one side by a focused laser beam and the light transmitted through the nanowire waveguide is then used to excite fluorophores on the exit side of the nanowaveguide. The excitation laser beam is circularly polarized to produce a cylindrically symmetrical illumination profile of the transmitted light emerging from the exit side of the nanowire waveguide.

As shown in our previous studies,^22,24^ strong resonant transmission of light through dielectric nanowire waveguides of sizes well below the diffraction limit is possible when the dielectric constants of the metal cladding and the dielectric core are matched, enabling resonant excitation of surface plasmons at the metal–dielectric boundary. Resonant transmission of 490 nm light through 40-nm-diameter cylindrical zinc oxide nanowires embedded in a silver film was demonstrated,^23^ and the transmission wavelengths can be tuned by varying the nanowire diameter.^25^ As the strong transmission is enabled by a resonant mechanism, the transmitted light is dominated by near fields emerging from the nanowaveguide exit and is strongly localized in both lateral and longitudinal directions. The lateral confinement is set by the nanowire diameter, while the transmitted light decays exponentially in the axial direction and only extends for about 10 nm, which is on the order of typical cell membrane thickness. Since resonant transmission of light through a nanowire waveguide of particular size is limited to a narrow band of wavelengths, the fluorescence signal at longer wavelengths cannot transmit efficiently back through the nanowire waveguide. To overcome this, a second objective lens confocal with the illumination spot created by the nanowaveguide is introduced to collect the fluorescence signal, as shown in Fig. 1.

## III. RESULTS AND DISCUSSION

### A. The NIFS illumination profile

**FIG. 2.**
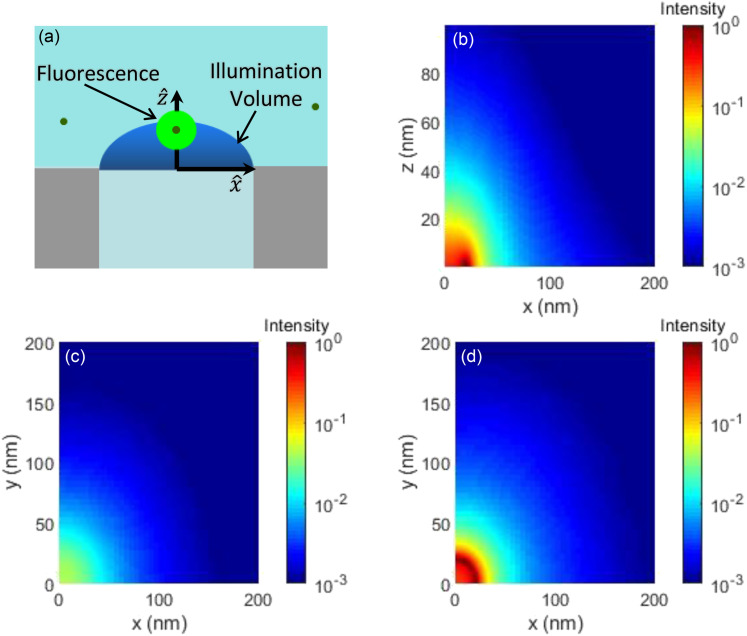
Cross sections of the NIFS intensity profile calculated by the finite element method. (a) Schematic of the nanowire waveguide device. (b) Intensity profile in the x–z plane showing its exponential decay along the z axis. (c) Intensity profile in the z = 23 nm plane showing it is nearly Gaussian in the lateral directions. (d) Intensity profile in the z = 2.3 nm plane near the nanowire waveguide exit surface showing it has an annular Gaussian profile centered around the edges of the waveguide.

When the nanowire waveguide is introduced producing a NIFS setup, the illumination profile $W\left(r\right)$ is radically altered, as shown in Fig. 2. Using the finite element method (COMSOL Wave Optics Module) and a simulation setup described in our previous work,^22^ we have determined the intensity profile generated by resonant transmission of 490 nm circularly polarized light through a cylindrical 40-nm-diameter zinc dioxide nanowire waveguide embedded in a 100-nm-thick silver film [see the inset of Figs. 1 and 2(a)]. As shown in Figs. 2(b) and 2(c), the new intensity decays exponentially along the z-axis as it gets further away from the terminal side of the nanowire device and has a nearly Gaussian profile in the lateral direction. This allows us to approximate the intensity profile as

$$W_{3-D}\left(r\right)\approx \left\{\begin{array}{cc} e^{-\frac{2\left(x^{2}+y^{2}\right)}{r_{0}^{2}}}e^{-\frac{z}{z_{0}}}\; & z>0\\ 0\; & z<0, \end{array}\right.$$ (5)

where *r*_0_ is the lateral extent of our illumination profile (∼60 nm) and *z*_0_ is the decay length along the z-axis (∼12 nm). We have taken the intensity profile to be zero for *z* < 0 because the fluorophores cannot enter the nanowire waveguide device.

However, due to the lightning-rod effect where electromagnetic fields tend to be enhanced outside metallic surfaces of sharp curvatures,^26^ the intensity rises rapidly at the right-angle edges of the nanowire near the waveguide exit surface [see Fig. 2(d)]. Thus, the illumination profile is no longer centered along the axis of the waveguide in the region near the nanowire exit surface. Instead, for 0 < *z* < 5 nm, we have found that the profile can be approximated as an annular Gaussian function centered around the edges of the waveguide,

$$W_{2-D}\left(r\right)\approx e^{-\frac{{\left(r-a_{0}\right)}^{2}}{b_{0}^{2}}},$$ (6)

where $r=\sqrt{x^{2}+y^{2}}$, *a*_0_ is the radius of the nanowire waveguide (∼20 nm), and *b*_0_ is the width of the central intensity peak (∼8 nm). These edge effects are due to the sharp curvature of the metal–dielectric boundary at the waveguide exit surface and thus do not extend very far beyond the terminus of the waveguide device (*z* < 5 nm), resulting in a negligible effect on the 3D illumination volume. The ACF for most 3D NIFS measurements can thus be calculated using Eq. (5). However, Eq. (6) needs to be used as the intensity profile to calculate the ACF when the movement of fluorophores is confined in a 2D surface near the nanowaveguide exit.

### B. Monte Carlo simulations

In order to verify the analytical ACFs for NIFS, we ran Monte Carlo simulations to simulate the behavior of fluorophores under various illumination conditions. We used the simulations to generate a simulated fluorescence signal and calculate what the simulated autocorrelation curve will look like. These autocorrelation data can then be fit with our ACF analytical forms to verify whether these expressions accurately describe the autocorrelations that one would get in actual experiments.

Following the recipe outlined in a previous study by Dix *et al.*,^27^ we accomplish each numerical simulation of conventional FCS and NIFS experiments in three independent stages. The first stage involves setting up the simulation geometry and generating the initial positions and velocities of fluorescent particles using a random number generator. In this step, we first define a rectangular simulation box and then define a sub-region of this space to be the observation volume where the laser intensity profile defined by Eq. (3) or the NIFS illumination profiles shown in Fig. 2 are introduced. Periodic boundary conditions are used for all sides of the simulation box except for the nanowaveguide exit surface in 3D NIFS simulations. For that, we have assumed a hard-wall boundary, meaning the axial momentum of molecules hitting the nanowaveguide exit surface is reversed, but their tangential momentum remains intact.

The second stage involves simulating the particle dynamics and collecting the fluorescence signal. The Brownian motion of the particles is simulated by assigning a random change in velocity to each particle. We note that the diffusive timescales considered in this study are much longer than the solvent viscous relaxation time *τ*_*υ*_ = *a*^2^/*υ*, where *a* is the particle radius and *υ* is the kinematic viscosity of the solvent,^28^ and the hydrodynamic response of the solvent occurring over the time scale of *τ*_*υ*_ can thus be neglected here. The fluorophores that are inside of the observation box are allowed to fluoresce based on Monte Carlo statistics and the laser intensity at their given positions. The photon arrival time and the corresponding fluorescence signal are then recorded based on the number of fluoresced fluorophores at that instant. A typical single run of our simulated experiments has a total of 10^7^ random walk steps for each molecule with a time step of 100 ns. In order to obtain sufficient statistics within these simulation times, we have adjusted the intensity amplitude of the exciting beam until the photon count rate of the fluorescence signal is on the order of 100 kHz. We note that in actual experiments the intensity of the exciting beam needs to be kept at reasonable levels to avoid photobleaching of the fluorescent particles.

In the last stage of the simulation, we use the photon arrival time and the fluorescence signal generated in the second step to correlate the fluorescence signal at every time step *t*_*i*_ with that at *t*_*j*_ = *t*_*i*_ + *τ*. By varying the delay time *τ*, we are able to generate our autocorrelation curve *G*(*τ*).

**FIG. 3.**
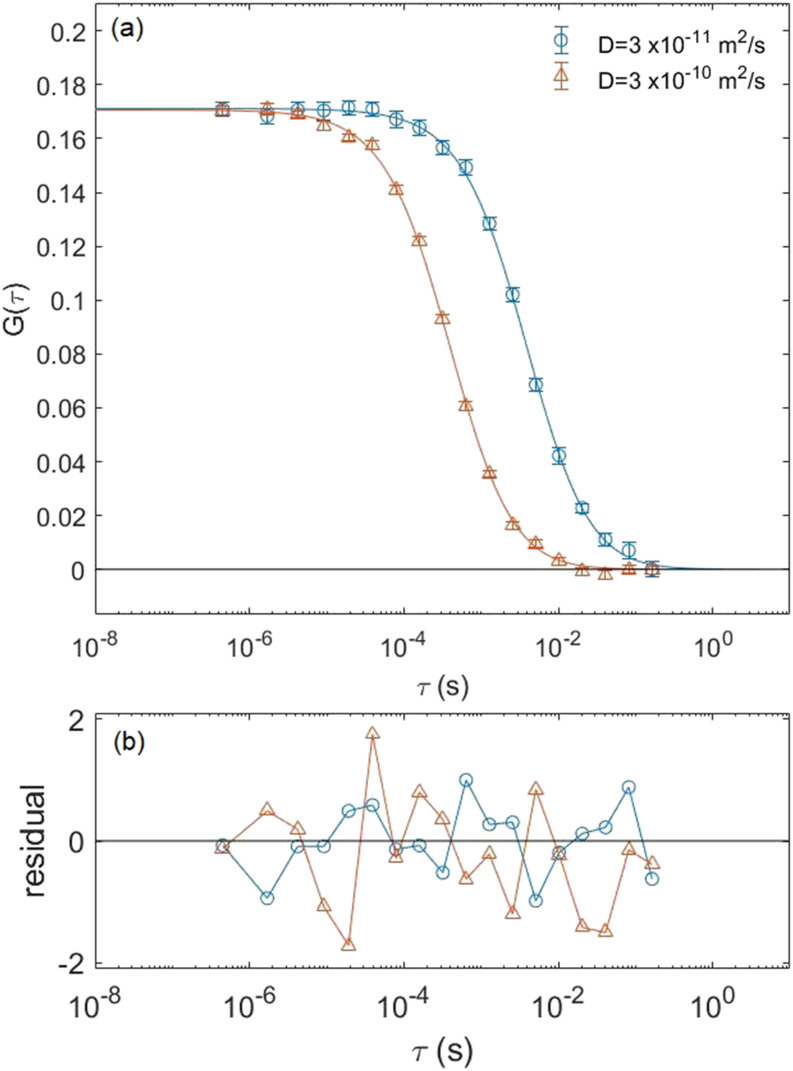
(a) Comparison of the simulated correlation curve (open circles) vs the analytical ACF (solid lines) for traditional FCS given by Eq. (4). The simulated data were created by averaging 20 runs of 1000 fluorophores each in a 1000-fL simulation box but with different random number seeds. These data were created for two different diffusion constants of 3 × 10^−11^ m^2^/s (circles) and 3 × 10^−10^ m^2^/s (triangles). (b) Residuals defined as the difference between the simulation data and the analytical ACF result normalized by the standard deviation at each data point vs the delay time.

We have validated our simulation codes with conventional FCS by generating simulated correlation data and comparing the results with the analytical ACF given by Eq. (4). We simulated the Brownian motion of 1000 simulated fluorophores for two diffusion constants, 3 × 10^−11^ and 3 × 10^−10^ m^2^/s, in a 1000-fL simulation box and averaged 20 runs with identical parameters but different seed values for the random number generator. The following parameters for the Gaussian laser beam were used in our simulation: *r*_0_ = 0.708 *µ*m and *κ* = 3. As we can see in Fig. 3, the simulations fit well to the analytical ACF for conventional FCS based on the small residuals between the data and the fits. From the fits, we can determine *τ*_*D*_ and *V*_eff_, which can be seen in Table I and are in excellent agreement with our input parameters.

**TABLE I.** Comparison of parameters calculated from input conditions vs parameters obtained from fits for traditional FCS.

	Input parameters		Fit parameters	
*D* (m^2^/s)	*τ*_*D*_ (ms)	*V*_eff_ (fL)	*τ*_*D*_ (ms)	*V*_eff_ (fL)
3 × 10^−11^	4.2	5.93	4.0	5.84
3 × 10^−10^	0.42	5.93	0.41	5.86

### C. NIFS for 3D samples

Next, we calculate and test the ACF for NIFS in the 3D case, where the illumination intensity profile is now given by Eq. (5). Additionally, since the nanowire device introduces a boundary condition at *z* = 0, the density autocorrelation term must be altered. Taking this and the new intensity profile into consideration, one can then use Eq. (2) to obtain the following ACF for NIFS, which resembles that found in TIR-FCS:^15^

$$\begin{array}{ll} G_{3-D}\left(\tau \right) & =\frac{1}{2V_{e\mathrm{ff}}\left\langle C\right\rangle }{\left(1+\frac{\tau }{\tau_{D}}\right)}^{-1}\\ & \;\times \left[\sqrt{\frac{\tau }{\pi \tau_{z}}}+\left(1-\frac{\tau }{2\tau_{z}}\right)erfcx\left(\sqrt{\frac{\tau }{4\tau_{z}}}\right)\right], \end{array}$$ (7)

where $V_{e\mathrm{ff}}=\pi r_{0}^{2}z_{0}$ is the effective focal volume, $\tau_{D}=\frac{r_{0}^{2}}{4D}$ is the characteristic diffusion time, $\tau_{z}=\frac{z_{0}^{2}}{4D}=\frac{z_{0}^{2}}{r_{0}^{2}}\tau_{D}$, and erfcx(*z*) = exp(*z*^2^)erfc(*z*) = exp(*z*^2^)[1 −erf(*z*)] is the scaled complementary error function.

Notice that this ACF, such as the ACF for traditional FCS, has a ${\left(1+\frac{\tau }{\tau_{D}}\right)}^{-1}$ term as a result of fluorophores freely diffusing under a Gaussian intensity in the lateral dimensions. The other term in the bracket is entirely due to diffusion in the axial direction where the intensity profile is decaying exponentially along the z-axis. We note that although the ACF expression for 3D NIFS resembles that in TIR-FCS, the deep-subwavelength confinement in both lateral and longitudinal directions will result in a much smaller sample volume than achieved by TIR-FCS.

**FIG. 4.**
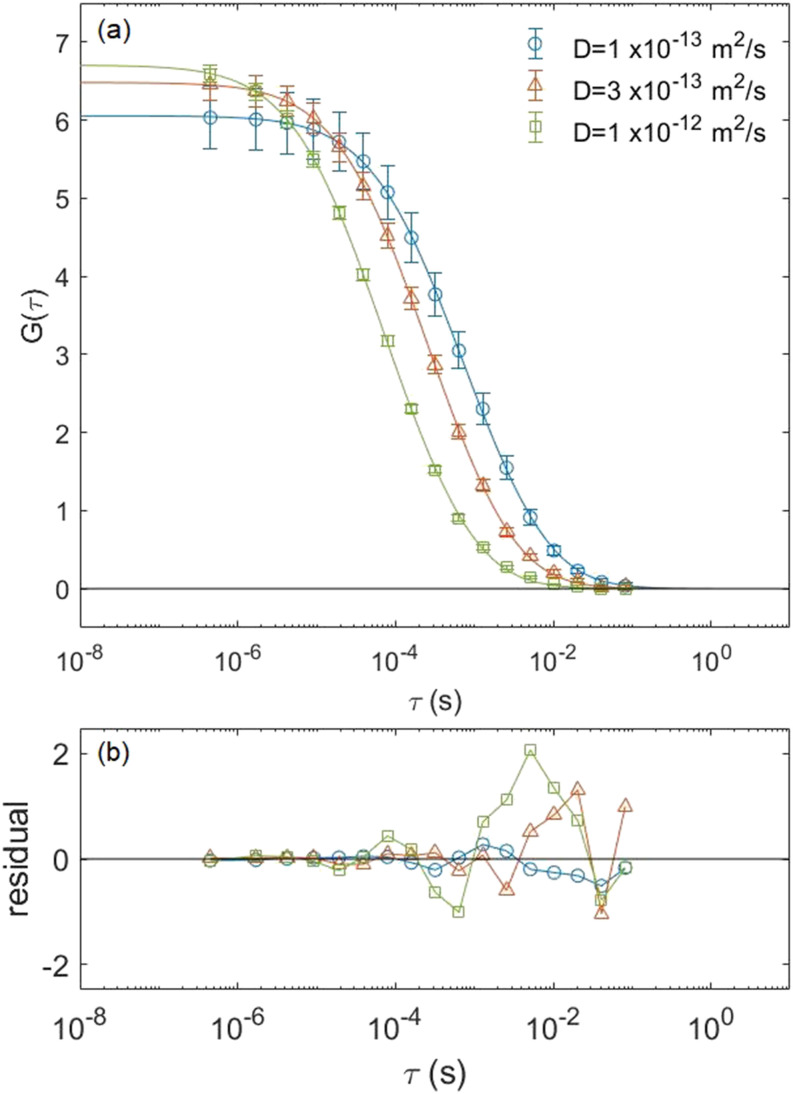
(a) Comparison of the simulated correlation curve for 3D NIFS (open circles) vs the analytical ACF (solid lines). The data were created by averaging 20 runs with identical parameters but different random number seeds for 1000 simulated fluorophores in a 2-fL simulation box. These data were created for three different diffusion constants of 1 × 10^−13^ m^2^/s (circles), 3 × 10^−13^ m^2^/s (triangles), and 1 × 10^−12^ m^2^/s (squares). (b) Residuals vs the delay time.

In order to verify the ACF we found in Eq. (7), we simulated particles undergoing Brownian motion in the laser focal region of a NIFS setup. To do this, we replaced the Gaussian intensity profile with the intensity distribution coming out of a nanowire waveguide calculated by the finite element method. We simulated the Brownian motion of 1000 simulated fluorophores for three diffusion constants, 1 × 10^−13^, 3 × 10^−13^, and 1 × 10^−12^ m^2^/s, in a 2-fL simulation box and averaged 20 runs with identical parameters but different seed values for the random number generator. The simulated correlation curve is then fitted to our analytical ACF given by Eq. (7) using fitting parameters *G*_0_ = 1/(2*V*_eff_⟨*C*⟩), *τ*_*D*_, and *τ*_*z*_. The result is shown in Fig. 4.

We found the majority of the points have a small residual of less than 1.0 validating the analytical ACF given by Eq. (7). The values for *τ*_*D*_ determined from the fit were in fairly good agreement with our expected values, as can be seen in Table II. In addition, the value of *V*_eff_ determined from the fit was also in agreement with our expected effective volume and is more than four orders of magnitude smaller than that in traditional FCS. The discrepancy between the fitting and expected values is not unreasonable given that the intensity is not exactly Gaussian near the waveguide exit surface due to edge effects. Our results indicate that NIFS is a viable technique suitable for probing samples with concentrations in the 10–100 *µ*M range.

**TABLE II.** Comparison of parameters calculated from input conditions vs parameters obtained from fits for 3D NIFS.

	Input parameters		Fit parameters	
*D* (m^2^/s)	*τ*_*D*_ (ms)	*V*_eff_ (zL)	*τ*_*D*_ (ms)	*V*_eff_ (zL)
1 × 10^−13^	9.0	136	10.2	165.1
3 × 10^−13^	3.0	136	3.8	154.3
1 × 10^−12^	0.9	136	1.3	149.2

### D. NIFS for 2D samples

Since the intensity profile decays exponentially along the axial direction, NIFS will be very suitable for studying the dynamics of molecules bound onto a 2D surface such as the cell membrane. When the fluorophores are confined to a 2D surface situated directly above the nanowaveguide exit, the ACF must be calculated using the intensity profile given by Eq. (6) in order to take into account the enhanced fields near the nanowaveguide edges. With these modifications, it is no longer possible to obtain an exact solution for the ACF. However, by considering the small- and large-timescales separately, we can still obtain an approximate result by using a piece-wise function (see the Appendix for derivation),

$$G_{2-D}\left(\tau \right)=\left\{\begin{array}{cc} G_{0}{\left(1+\frac{\tau }{\tau_{D}}\right)}^{-1}\; & \tau \leq \tau_{crit}\\ G_{0}{\left(4\pi \frac{\tau_{C}}{\tau }\frac{\tau_{D}}{\tau }\right)}^{\frac{1}{2}}\left[1+{\left(\frac{\tau_{C}}{\tau }\right)}^{2}+\frac{1}{4}{\left(\frac{\tau_{C}}{\tau }\right)}^{4}\right]e^{-\frac{2\tau_{C}}{\tau }}\; & \tau >\tau_{crit}, \end{array}\right.$$ (8)

where $\tau_{D}=\frac{b_{0}^{2}}{2D}$, $\tau_{C}=\frac{a_{0}^{2}}{4D}$, and $G_{0}={\left(A_{e\mathrm{ff}}\left\langle C\right\rangle \right)}^{-1}={\left[{\left(2\pi \right)}^{\frac{3}{2}}a_{0}b_{0}\left\langle C\right\rangle \right]}^{-1}$ are fitting parameters and *τ*_crit_ is some critical time separating the two timescales and is on the order of *τ*_*C*_. Notice that in the small-timescale *τ* ≤ *τ*_crit_, the ACF is identical to that for a one-dimensional Gaussian intensity profile. This is because at these timescales the particles will only diffuse through length scales on the order of the width of the radial Gaussian peak *b*_0_. At the large-timescale *τ* > *τ*_crit_, the correlation function is much more complicated and contains no features corresponding to traditional Gaussian intensities because at these timescales the fluorophores will sample the entire donut-shaped intensity profile in the 2D plane.

In order to verify the ACF for 2D NIFS we found in Eq. (8), we introduced a 2D membrane to be situated directly above the nanowaveguide exit plane. Specifically, we created a second set of simulated fluorophores whose motion is restricted to the horizontal plane at *z* = 2.3 nm above the nanowire waveguide surface. We simulated the Brownian motion of 400 simulated fluorophores with three diffusion constants, 1 × 10^−13^, 3 × 10^−13^, and 1 × 10^−12^ m^2^/s on a 4 *µ*m^2^ surface area and averaged 20 runs with identical parameters but different seed values for the random number generator.

**FIG. 5.**
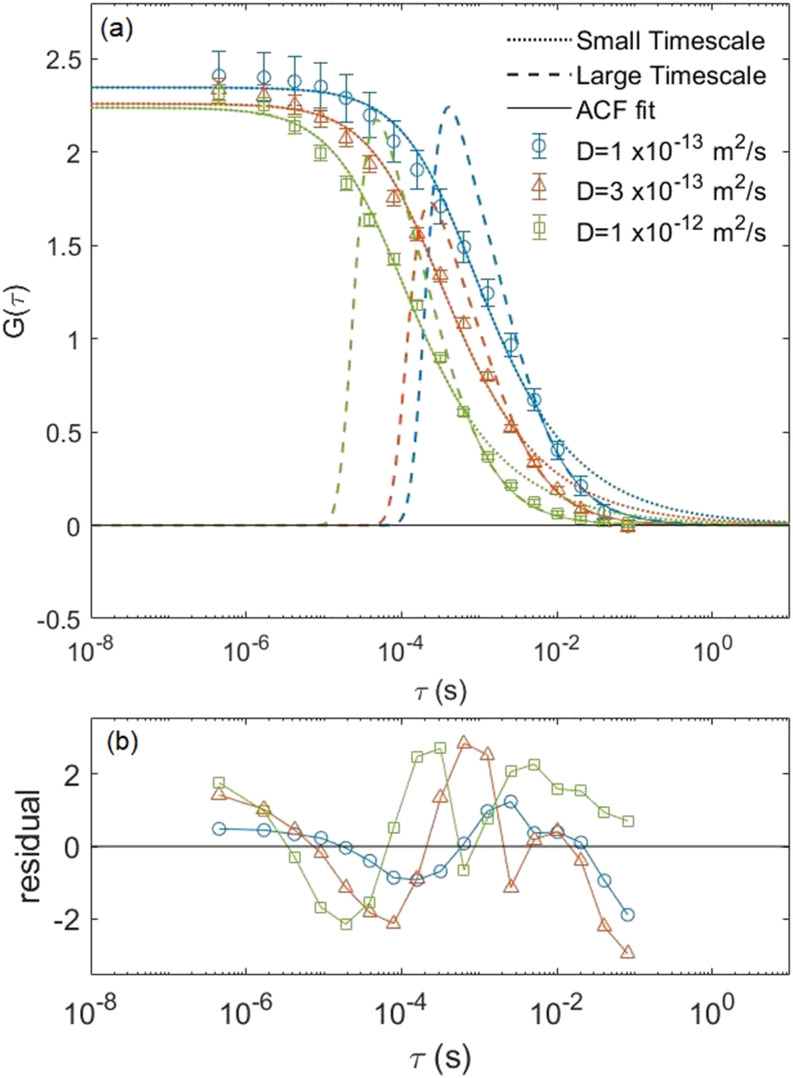
(a) Comparison of the simulated correlation curve for 2D NIFS (open circles) vs the two-component analytical ACF (solid lines) given by Eq. (8) for 2D NIFS. The data were created by averaging 20 runs with identical parameters but different random number seeds for 400 simulated fluorophores on a 4 *µ*m^2^ surface area. Note that the small- (dotted lines) and large- (dashed lines) timescale fit functions intersect at ***τ*** ∼ 8***τ***_***C***_. These data were created for three different diffusion constants of 1 × 10^−13^ m^2^/s (circles), 3 × 10^−13^ m^2^/s (triangles), and 1 × 10^−12^ m^2^/s (squares). (b) Residuals vs the delay time.

We noticed that the photon count rate was much lower for the 2D simulations than that found in 3D NIFS simulations. This was likely due to the fact that our membrane results in a much smaller sample volume than in the 3D case, so similar concentrations meant fewer molecules being excited. To overcome this problem, we increased the intensity amplitude of the exciting beam until the photon count rate of the fluorescence signal was comparable to the rates we were getting in the 3D simulations. We then fit the simulated correlation curve with the analytical ACF for 2D NIFS given by Eq. (8). The results are shown in Fig. 5 along with the two components of the fit function.

We can see that the small-timescale component (dotted lines) of the ACF fits the simulation data very well except for times above the critical time *τ*_crit_. On the other hand, the large-timescale approximation (dashed lines) of the ACF fits the data well for timescales greater than the critical time but fails to describe the movement of molecules on the short timescales. From the fit, we determined that the two characteristic diffusion times *τ*_*D*_ and *τ*_*C*_ are in relatively good agreement with our expected values based on our input parameters (see Table III). We also determined the effective sampling area *A*_eff_ to be consistent with our expected value based on the input parameters.

**TABLE III.** Comparison of parameters calculated from input conditions vs parameters obtained from fits for 2D NIFS.

	Input parameters			Fit parameters		
*D* (m^2^/s)	*τ*_*D*_ (ms)	*τ*_*C*_ (ms)	*A*_eff_ (μm^2^)	*τ*_*D*_ (ms)	*τ*_*C*_ (ms)	*A*_eff_ (μm^2^)
1 × 10^−13^	0.32	1.0	2.52 × 10^−3^	0.43	0.68	4.3 × 10^−3^
3 × 10^−13^	0.11	0.33	2.52 × 10^−3^	0.16	0.39	4.4 × 10^−3^
1 × 10^−12^	32 *µ*s	0.1	2.52 × 10^−3^	52 *µ*s	0.08	4.5 × 10^−3^

The discrepancy between the fitting and expected values for effective area is not unreasonable given that the annular Gaussian function we used to derive the analytical ACF for 2D NIFS is only an approximation of the actual intensity profile. The effective sample area of about 4.0 × 10^−3^ *µ*m^2^ is well below the diffraction limit and can be advantageous for the study of densely distributed membrane proteins that typically move with diffusion coefficients of 1 *µ*m^2^/s or smaller with varying local concentrations on the cell membrane.^29^ Since simultaneous excitation of multiple molecules will be limited in 2D NIFS even for a fluorophore density of as high as 250 particles/*μ*m^2^, the technique also has the benefit of minimizing photobleaching and self-quenching of fluorophores in studying membrane proteins.

Compared with TIR-FCS that provides subwavelength confinement in the axial direction but no confinement in the lateral direction, the NIFS technique has the advantage of deep-subwavelength confinement in both lateral and axial directions and can be advantageous in studying highly concentrated molecules confined on a 2D plane such as the cell membrane. With its sub-50-nm lateral confinement, STED-FCS can be a useful tool to study membrane-bound biomolecules at high concentration conditions. However, due to its poor axial confinement, the STED-FCS technique will not be able to exclude signal from fluorescent molecules diffusing near the cell membrane in the cytosol. On the other hand, with its 10-nm axial confinement, NIFS will only measure signal from membrane-bound molecules and can be useful in studying the binding dynamics of molecules to the cell membrane.

We note that illumination volumes in the zeptoliter range have also been achieved with other near-field-based approaches using optical nanostructures such as zero-mode waveguides^30,31^ and plasmonic devices.^32^ However, the structures used in these approaches often have nanoscale curvatures and are not suitable for studying cell membranes as the membrane surface will be forced to conform to the device boundaries, resulting in strong disturbance to the samples. In the NIFS approach, the flat surface at the exit side of the nanowaveguide will cause minimal disturbance to the cell and is thus suitable for studying the dynamics of membrane proteins in the physiologically relevant concentration range.

## IV. CONCLUSION

In this work, we have introduced a nanowaveguide-illumination-based method to reduce the sample volume well below the diffraction limit encountered in conventional FCS. By utilizing the strongly confined near fields emerging from the exit of a nanowire waveguide enabled by a resonant transmission mechanism, this nanowaveguide-illuminated fluorescence correlation spectroscopy (NIFS) technique allows for the creation of a nanoscopic illumination profile with deep-subwavelength confinement in both lateral and axial directions. Utilizing the illumination intensity profile obtained from finite element method simulations, we have obtained the autocorrelation functions for 3D and 2D NIFS and validated these expressions using Monte Carlo simulations. Our results reveal an illumination volume four orders of magnitude smaller than the diffraction limit, which would in turn enable the study of biological processes at the single molecule level in the 10–100 *µ*M concentration range. The deep-subwavelength confinement in the axial direction and its flat surface geometry also make the NIFS technique well suited for the study of 2D surfaces with potential application in studying the dynamics of protein molecules on cell membranes. As the majority of therapeutics target membrane proteins, the NIFS technique may find its use in high-throughput screening of drug candidates in pharmacology studies.

## Data Availability

The data that support the findings of this study are available from the corresponding author upon reasonable request.

## APPENDIX: DERIVATION OF THE 2D NIFS AUTOCORRELATION FUNCTION

Our starting point for calculating the 2D NIFS ACF is with Eq. (2),

$$G\left(\tau \right)=\frac{\int d^{3}r\int d^{3}r^{\prime }W\left(r\right)W\left(r^{\prime }\right)\left\langle \delta C\left(r,0\right)\delta C\left(r^{\prime },\tau \right)\right\rangle }{{\left(\left\langle C\right\rangle \int d^{3}rW\left(r\right)\right)}^{2}},$$ (A1)

where *W*(***r***) is the spatial intensity distribution and $\left\langle \delta C\left(r,0\right)\delta C\left(r^{\prime },\tau \right)\right\rangle$ is the density autocorrelation function. The 2D density correlation function is given by

$$\left\langle \delta C\left(r,0\right)\delta C\left(r^{\prime },\tau \right)\right\rangle =\left\langle C\right\rangle e^{-\frac{{\left(r-r^{\prime }\right)}^{2}}{4D\tau }}{\left(4\pi D\tau \right)}^{-1}.$$ (A2)

Our 2D intensity distribution found in Eq. (6) is

$$W\left(r\right)=e^{-\frac{{\left(r-a_{0}\right)}^{2}}{b_{0}^{2}}},$$ (A3)

with *a*_0_ being the radial location of the Gaussian peak and *b*_0_ being the Gaussian width. For a typical NIFS setup, we have *a*_0_ ≈ 20 nm and *b*_0_ ≈ 8 nm.

Substituting Eqs. (A2) and (A3) into Eq. (A1) and performing the integration in 2D, we have

$$G\left(\tau \right)=\frac{1}{4\pi D\tau \left\langle C\right\rangle }\frac{\int \int e^{-\frac{{\left(r-a_{0}\right)}^{2}}{b_{0}^{2}}}e^{-\frac{{\left(r^{\prime }-a_{0}\right)}^{2}}{b_{0}^{2}}}e^{-\frac{{\left(r-r^{\prime }\right)}^{2}}{4D\tau }}d^{2}rd^{2}r^{\prime }}{{\left(\int e^{-\frac{{\left(r-a_{0}\right)}^{2}}{b_{0}^{2}}}d^{2}r\right)}^{2}}.$$ (A4)

The integral in the denominator can be evaluated to be

$$\int e^{-\frac{{\left(r-a_{0}\right)}^{2}}{b_{0}^{2}}}d^{2}r=2\pi \left\{-\frac{1}{2}\pi^{\frac{1}{2}}a_{0}b_{0}\left[Q\left(-\frac{1}{2},\frac{a_{0}^{2}}{b_{0}^{2}}\right)-2\right]\right\},$$ (A5)

where *Q*(*a*, *ξ*) is the regularized gamma function and is given by *Q*(*a*, *ξ*) = *Γ*(*a*, *ξ*)/*Γ*(*a*). For *ξ* > 1, the regularized gamma function approaches zero. Since $\frac{a_{0}^{2}}{b_{0}^{2}}∼6$, we have $Q\left(-\frac{1}{2},\frac{a_{0}^{2}}{b_{0}^{2}}\right)\to 0$ in Eq. (A5) and obtain

$$\int e^{-\frac{{\left(r-a_{0}\right)}^{2}}{b_{0}^{2}}}d^{2}r=2\pi^{\frac{3}{2}}a_{0}b_{0},$$ (A6)

$$\begin{array}{ll} G\left(\tau \right) & ={\left(16\pi^{4}D\tau \left\langle C\right\rangle a_{0}^{2}b_{0}^{2}\right)}^{-1}\int \int e^{-\frac{{\left(r-a_{0}\right)}^{2}}{b_{0}^{2}}}e^{-\frac{{\left(r^{\prime }-a_{0}\right)}^{2}}{b_{0}^{2}}}e^{-\frac{{\left(r-r^{\prime }\right)}^{2}}{4D\tau }}d^{2}rd^{2}r^{\prime }\\ & =\frac{G_{0}}{{\left(2\pi \right)}^{\frac{5}{2}}D\tau a_{0}b_{0}}I, \end{array}$$ (A7)

where $G_{0}={\left[\left\langle C\right\rangle {\left(2\pi \right)}^{\frac{3}{2}}a_{0}b_{0}\right]}^{-1}$ and

$$I=\int \int e^{-\frac{{\left(r-a_{0}\right)}^{2}}{b_{0}^{2}}}e^{-\frac{{\left(r^{\prime }-a_{0}\right)}^{2}}{b_{0}^{2}}}e^{-\frac{{\left(r-r^{\prime }\right)}^{2}}{4D\tau }}d^{2}rd^{2}r^{\prime }.$$ (A8)

In order to evaluate the integral in Eq. (A8), we introduce two new variables *x* = *r* − *a*_0_ and *x*′ = *r*′ − *a*_0_, both on the order of *b*_0_. The integral then becomes

$$\begin{array}{ll} I & =\int_{-a_{0}}^{\infty }\int_{0}^{2\pi }dxd\phi \int_{-a_{0}}^{\infty }\int_{0}^{2\pi }dx^{\prime }d\phi^{\prime }\cdot \left(x+a_{0}\right)\left(x^{\prime }+a_{0}\right)\\ & \;\times e^{-\frac{x^{2}+{x^{\prime }}^{2}}{b_{0}^{2}}\left(1+\frac{b_{0}^{2}}{4D\tau }\right)}e^{2\frac{xx^{\prime }}{4D\tau }}e^{-\frac{2\left[a_{0}^{2}+a_{0}\left(x+x^{\prime }\right)+xx^{\prime }\right]\left(1-\cos\phi^{\prime }\right)}{4D\tau }}. \end{array}$$ (A9)

At this point, we introduce another new variable $a=\frac{2\left[a_{0}^{2}+a_{0}\left(x+x^{\prime }\right)+xx^{\prime }\right]}{4D\tau }=\frac{2rr^{\prime }}{4D\tau }$, which gives us

$$\begin{array}{ll} I & =\int_{-a_{0}}^{\infty }\int_{0}^{2\pi }dxd\phi \int_{-a_{0}}^{\infty }dx^{\prime }\cdot \left(x+a_{0}\right)\left(x^{\prime }+a_{0}\right)\\ & \;\times e^{-\frac{x^{2}+{x^{\prime }}^{2}}{b_{0}^{2}}\left(1+\frac{b_{0}^{2}}{4D\tau }\right)}e^{2\frac{xx^{\prime }}{4D\tau }}e^{-a}\int_{0}^{2\pi }d\phi^{\prime }e^{a\cos\phi^{\prime }}. \end{array}$$ (A10)

This integral cannot be evaluated in the given form. However, we can solve if we insert approximations for *a* based on the timescale *τ*.

### 1. The small-timescale approximation

For timescales *τ* smaller than some critical value *τ*_crit_ ∼ *τ*_*C*_ where $\tau_{C}\equiv \frac{a_{0}^{2}}{4D}$ is the characteristic time that fluorophores diffuse across the entire annular illumination profile, we expect $a=\frac{2\left[a_{0}^{2}+a_{0}\left(x+x^{\prime }\right)+xx^{\prime }\right]}{4D\tau }=\frac{2\tau_{c}}{\tau }\left(1+\frac{x}{a_{0}}+\frac{x^{\prime }}{a_{0}}+\frac{xx^{\prime }}{a_{0}^{2}}\right)>1$, in which case, the main contribution to the integral $e^{-a}\int_{0}^{2\pi }e^{-a\left(1-\cos\phi \right)}d\phi$ in Eq. (A10) comes from small values of *ϕ* such that *a*(1 − cos *ϕ*) ≤ 1. The integral can then be approximated as

$$\begin{array}{ll} \int_{0}^{2\pi }e^{-a\left(1-\cos\phi \right)}d\phi & \approx \int_{-\pi }^{\pi }e^{-a\frac{\phi^{2}}{2}}d\phi \\ & \approx \sqrt{\frac{2\pi }{a}}=\sqrt{\frac{\pi \tau }{\tau_{C}}}{\left(1+\frac{x}{a_{0}}+\frac{x^{\prime }}{a_{0}}+\frac{xx^{\prime }}{a_{0}^{2}}\right)}^{-\frac{1}{2}}. \end{array}$$ (A11)

Introducing $u\equiv \frac{x}{a_{0}}$ and $u^{\prime }\equiv \frac{x^{\prime }}{a_{0}}$ and keeping up to first-order terms in *u* and *u*′, we can rewrite the above results as

$$e^{-a}\int_{0}^{2\pi }e^{a\cos\phi^{\prime }}d\phi^{\prime }\approx \sqrt{\frac{\pi \tau }{\tau_{C}}}\left(1-\frac{u}{2}-\frac{u^{\prime }}{2}\right).$$ (A12)

Substituting the above result into Eq. (A10), we obtain

$$\begin{array}{ll} I & \approx 2\pi a_{0}^{4}\sqrt{\frac{\pi \tau }{\tau_{C}}}\int_{-1}^{\infty }du\int_{-1}^{\infty }du^{\prime }\left(1+u\right)\left(1+u^{\prime }\right)\left(1-\frac{u}{2}-\frac{u^{\prime }}{2}\right)\\ & \;\times e^{-\beta \left(u^{2}+u^{\prime 2}\right)+\frac{2\tau_{C}}{\tau }uu^{\prime }}, \end{array}$$ (A13)

where $\beta =\left(a_{0}^{2}/b_{0}^{2}\right)\left(1+b_{0}^{2}/4D\tau \right)$. Since $\frac{a_{0}^{2}}{b_{0}^{2}}\gg 1$, large values of *u* and *u*′ will not contribute much to the integral due to the exponential decay functions in the integrand, and we can then approximate the lower bound of the integral in Eq. (A13) to be −∞ and obtain

$$\begin{array}{ll} I & \approx 2\pi^{\frac{3}{2}}a_{0}^{4}\sqrt{\frac{\tau }{\tau_{C}}}\int_{-\infty }^{\infty }\int_{-\infty }^{\infty }dudu^{\prime }\left(1+\frac{u}{2}+\frac{u^{\prime }}{2}\right)e^{-\beta \left(u^{2}+u^{\prime 2}\right)+\frac{2\tau_{C}}{\tau }uu^{\prime }}\\ \; & =2\pi^{\frac{3}{2}}a_{0}^{4}\sqrt{\frac{\tau }{\tau_{C}}}\int_{-\infty }^{\infty }\int_{-\infty }^{\infty }dudu^{\prime }\left(1+u^{\prime }\right)e^{-\beta \left(u^{2}+u^{\prime 2}\right)+\frac{2\tau_{C}}{\tau }uu^{\prime }}\\ \; & =2\pi^{\frac{3}{2}}a_{0}^{4}\sqrt{\frac{\pi \tau }{\beta \tau_{C}}}\int_{-\infty }^{\infty }du^{\prime }\left(1+u^{\prime }\right)e^{-\left(\beta -\frac{\tau_{C}^{2}}{\beta \tau^{2}}\right){u^{\prime }}^{2}}\\ \; & =2\pi^{\frac{5}{2}}a_{0}^{4}\sqrt{\frac{\tau }{\tau_{C}}}{\left(\frac{a_{0}^{4}}{b_{0}^{4}}+2\frac{a_{0}^{2}}{b_{0}^{2}}\frac{\tau_{C}}{\tau }\right)}^{-1/2}\\ \; & ={\left(2\pi \right)}^{\frac{5}{2}}a_{0}b_{0}D\tau {\left(1+\frac{\tau }{\tau_{D}}\right)}^{-\frac{1}{2}}, \end{array}$$ (A14)

where $\tau_{D}\equiv \frac{b_{0}^{2}}{2D}$ is the characteristic time that fluorophores diffuse through the width *b*_0_ of the radial Gaussian intensity profile. We have also used the symmetry between *u* and *u*′ in the second step of Eq. (A14).

Substituting the result in Eq. (A14) into Eq. (A7), we then obtain the following ACF for small-timescales *τ* < *τ*_crit_:

$$G\left(\tau \right)=G_{0}{\left(1+\frac{\tau }{\tau_{D}}\right)}^{-\frac{1}{2}},$$ (A15)

where $G_{0}={\left(\left\langle C\right\rangle A_{e\mathrm{ff}}\right)}^{-1}$ with the effective sample area defined as $A_{e\mathrm{ff}}={\left(2\pi \right)}^{\frac{3}{2}}a_{0}b_{0}$. Note that Eq. (A15) is the exact ACF one would get for fluorophores diffusing through a 1D Gaussian profile. This is because at short timescales, fluorophores diffuse through distances on the order of the width of the radial Gaussian peak and do not experience the global shape of the annular intensity profile.

### 2. The large-timescale approximation

For large-timescales *τ* > *τ*_crit_ ∼ *τ*_*C*_, we expect $a\approx \frac{2\tau_{C}}{\tau }\left(1+u+u^{\prime }\right)$ to be smaller than 1, and the integral $\int_{0}^{2\pi }e^{a\cos\phi^{\prime }}d\phi^{\prime }$ in Eq. (A10) can be evaluated using the following series:

$$\int_{0}^{2\pi }e^{a\cos\phi^{\prime }}d\phi^{\prime }=2\pi \overset{\infty }{\underset{n=0}{\sum }}\frac{{\left(\frac{a^{2}}{4}\right)}^{n}}{{\left(n!\right)}^{2}}.$$ (A16)

For values of *a* less than 1, we find this series converge very quickly and it is sufficient to approximate it using the first three terms (the relative error is less than 10^−3^). Keeping up to first-order terms in *u* and *u*′, we can then approximate the series in Eq. (A16) to be

$$\int_{0}^{2\pi }e^{a\cos\phi^{\prime }}d\phi^{\prime }=2\pi \left(1+\frac{a^{2}}{4}+\frac{a^{4}}{64}\right)\approx 2\pi \left[\eta +\gamma \left(u+u^{\prime }\right)\right],$$ (A17)

where $\eta =1+\frac{\tau_{C}^{2}}{\tau^{2}}+\frac{\tau_{C}^{4}}{4\tau^{4}}$ and $\gamma =2\frac{\tau_{C}^{2}}{\tau^{2}}+\frac{\tau_{C}^{4}}{\tau^{4}}$.

Substituting the above result into Eq. (A10), we obtain

$$\begin{array}{ll} I & \approx 2\pi^{2}a_{0}^{4}e^{-\frac{2\tau_{C}}{\tau }}\int_{-1}^{\infty }du\int_{-1}^{\infty }\left(1+u\right)\left(1+u^{\prime }\right)\\ & \;\cdot \left[\eta +\gamma \left(u+u^{\prime }\right)\right]e^{-\beta \left(u^{2}+u^{\prime 2}\right)+\frac{2\tau_{C}}{\tau }uu^{\prime }-\frac{2\tau_{C}}{\tau }\left(u+u^{\prime }\right)}. \end{array}$$ (A18)

Again, since large values of *u* and *u*′ will not contribute much to the integral due to the exponential decay functions in the integrand, we can then approximate the lower bound of the integral to be −∞ and obtain

$$\begin{array}{ll} I & \approx 4\pi^{2}a_{0}^{4}e^{-\frac{2\tau_{C}}{\tau }}\int_{\infty }^{\infty }\int_{-\infty }^{\infty }dudu^{\prime }\left[\eta +\left(\eta +\gamma \right)\left(u+u^{\prime }\right)\right]e^{-\beta \left(u^{2}+u^{\prime 2}\right)+\frac{2\tau_{c}}{\tau }uu^{\prime }-\frac{2\tau_{C}}{\tau }\left(u+u^{\prime }\right)}\\ \; & =4\pi^{2}a_{0}^{4}e^{-\frac{2\tau_{C}}{\tau }}\int_{\infty }^{\infty }\int_{-\infty }^{\infty }dudu^{\prime }\left[\eta +2\left(\eta +\gamma \right)u^{\prime }\right]e^{-\beta \left(u^{2}+u^{\prime 2}\right)+\frac{2\tau_{c}}{\tau }uu^{\prime }-\frac{2\tau_{C}}{\tau }\left(u+u^{\prime }\right)}\\ \; & =4\pi^{2}a_{0}^{4}e^{-\frac{2\tau_{C}}{\tau }}\sqrt{\frac{\pi }{\beta }}\int_{\infty }^{\infty }du^{\prime }\left[\eta +2\left(\eta +\gamma \right)u^{\prime }\right]e^{-\left(\beta -\frac{\tau_{C}^{2}}{\beta \tau^{2}}\right)u^{\prime 2}-2\left(\frac{\tau_{C}}{\tau }+\frac{\tau_{C}^{2}}{\beta \tau^{2}}\right)u^{\prime }+\frac{\tau_{C}^{2}}{\beta \tau^{2}}}\\ \; & =4\pi^{3}a_{0}^{4}e^{-\frac{2\tau_{C}}{\tau }}e^{-\frac{\tau_{C}^{2}}{\beta \tau^{2}}+\frac{\frac{\tau_{C}^{2}}{\beta \tau^{2}}{\left(\beta \tau +\tau_{C}\right)}^{2}}{\beta^{2}\tau^{2}-\tau_{C}^{2}}}{\left(\beta^{2}-\frac{\tau_{C}^{2}}{\tau^{2}}\right)}^{-\frac{1}{2}}\left[\eta +2\left(\eta +\gamma \right)\frac{\beta \tau \tau_{C}+\tau_{C}^{2}}{\beta^{2}\tau^{2}-\tau_{C}^{2}}\right]\\ \; & \approx 4\pi^{3}a_{0}^{2}b_{0}^{2}{\left(1+\frac{b_{0}^{2}}{4D\tau }\right)}^{-1}e^{-\frac{2\tau_{C}}{\tau }\left(1-\frac{b_{0}^{2}}{4D\tau }\right)}\left[1+\frac{\tau_{C}^{2}}{\tau^{2}}+\frac{\tau_{C}^{4}}{4\tau^{4}}+2\left(1+\frac{3\tau_{C}^{2}}{\tau^{2}}+\frac{5\tau_{C}^{4}}{4\tau^{4}}\right)\frac{b_{0}^{2}}{4D\tau }\right]. \end{array}$$ (A19)

We have used the symmetry between *u* and *u*′ in the second step of Eq. (A19). Since we have *β* ≫ 1, we have dropped *β*^−2^ and higher-order terms in the last step. Also, at large-timescales, we have $\tau >\tau_{C}\gg \frac{b_{0}^{2}}{4D}$; thus, we have used $\frac{\tau_{C}}{\beta \tau }=\frac{b_{0}^{2}}{4D\tau }{\left(1+\frac{b_{0}^{2}}{4D\tau }\right)}^{-1}\approx \frac{b_{0}^{2}}{4D\tau }$ in the last step. Substituting the result in Eq. (A19) into Eq. (A7) and expressing the geometry dimension parameters *a*_0_ and *b*_0_ in terms of the characteristic diffusion times $\tau_{C}\equiv \frac{a_{0}^{2}}{4D}$ and $\tau_{D}\equiv \frac{b_{0}^{2}}{2D}$, we then obtain

$$\begin{array}{ll} G\left(\tau \right) & =2\pi^{\frac{1}{2}}G_{0}{\left(\frac{\tau_{D}}{\tau }\right)}^{\frac{1}{2}}{\left(\frac{\tau_{C}}{\tau }\right)}^{\frac{1}{2}}{\left(1+\frac{\tau_{D}}{2\tau }\right)}^{-1}e^{-\frac{2\tau_{C}}{\tau }\left(1-\frac{\tau_{D}}{2\tau }\right)}\\ & \;\cdot \left[1+\frac{\tau_{C}^{2}}{\tau^{2}}+\frac{\tau_{C}^{4}}{4\tau^{4}}+\left(1+\frac{3\tau_{C}^{2}}{\tau^{2}}+\frac{5\tau_{C}^{4}}{4\tau^{4}}\right)\frac{\tau_{D}}{\tau }\right]. \end{array}$$ (A20)

Since we have $\frac{\tau_{D}}{\tau }\ll 1$, the ACF for large-timescales *τ* > *τ*_crit_ can be approximated as

$$G\left(\tau \right)\approx 2\pi^{\frac{1}{2}}G_{0}{\left(\frac{\tau_{D}}{\tau }\right)}^{\frac{1}{2}}{\left(\frac{\tau_{C}}{\tau }\right)}^{\frac{1}{2}}\left(1+\frac{\tau_{C}^{2}}{\tau^{2}}+\frac{\tau_{C}^{4}}{4\tau^{4}}\right)e^{-\frac{2\tau_{C}}{\tau }}.$$ (A21)

Combining the results for small- and large-timescales, we obtain our final form of the ACF for 2D NIFS as

$$G_{2-D}\left(\tau \right)=\left\{\begin{array}{cc} G_{0}{\left(1+\frac{\tau }{\tau_{D}}\right)}^{-1}\; & \tau \leq \tau_{crit}\\ G_{0}{\left(4\pi \frac{\tau_{C}}{\tau }\frac{\tau_{D}}{\tau }\right)}^{\frac{1}{2}}\left[1+{\left(\frac{\tau_{C}}{\tau }\right)}^{2}+\frac{1}{4}{\left(\frac{\tau_{C}}{\tau }\right)}^{4}\right]e^{-\frac{2\tau_{C}}{\tau }}\; & \tau >\tau_{crit}, \end{array}\right.$$ (A22)

where $G_{0}={\left(\left\langle C\right\rangle A_{e\mathrm{ff}}\right)}^{-1}$, $\tau_{D}\equiv \frac{b_{0}^{2}}{2D}$, and $\tau_{C}\equiv \frac{a_{0}^{2}}{4D}$ are fitting parameters. The critical time that separates the two results, *τ*_crit_, is on the order of *τ*_*C*_. A numerical analysis of the integral $e^{-a}\int_{0}^{2\pi }e^{a\cos\phi^{\prime }}d\phi^{\prime }$ shows that both approximations discussed above yield results with an error of less than 15% for values of *a* ranging from 0.25 to 3. Given the simple analytical form of the small-timescale result, it will be advantageous to use it for values of *a* down to 0.25 in fitting correlation data. This corresponds to a value of 8*τ*_*C*_ for *τ*_crit_.
